# SIFTS: updated Structure Integration with Function, Taxonomy and Sequences resource allows 40-fold increase in coverage of structure-based annotations for proteins

**DOI:** 10.1093/nar/gky1114

**Published:** 2018-11-16

**Authors:** Jose M Dana, Aleksandras Gutmanas, Nidhi Tyagi, Guoying Qi, Claire O’Donovan, Maria Martin, Sameer Velankar

**Affiliations:** 1 Protein Data Bank in Europe, European Molecular Biology Laboratory, European Bioinformatics Institute (EMBL-EBI), Wellcome Genome Campus, Hinxton, Cambridge CB10 1SD, UK; 2 Protein Function Development, European Molecular Biology Laboratory, European Bioinformatics Institute (EMBL-EBI), Wellcome Genome Campus, Hinxton, Cambridge CB10 1SD, UK; 3 Metabolomics, European Molecular Biology Laboratory, European Bioinformatics Institute (EMBL-EBI), Wellcome Genome Campus, Hinxton, Cambridge CB10 1SD, UK

## Abstract

The Structure Integration with Function, Taxonomy and Sequences resource (SIFTS; http://pdbe.org/sifts/) was established in 2002 and continues to operate as a collaboration between the Protein Data Bank in Europe (PDBe; http://pdbe.org) and the UniProt Knowledgebase (UniProtKB; http://uniprot.org). The resource is instrumental in the transfer of annotations between protein structure and protein sequence resources through provision of up-to-date residue-level mappings between entries from the PDB and from UniProtKB. SIFTS also incorporates residue-level annotations from other biological resources, currently comprising the NCBI taxonomy database, IntEnz, GO, Pfam, InterPro, SCOP, CATH, PubMed, Ensembl, Homologene and automatic Pfam domain assignments based on HMM profiles. The recently released implementation of SIFTS includes support for multiple cross-references for proteins in the PDB, allowing mappings to UniProtKB isoforms and UniRef90 cluster members. This development makes structure data in the PDB readily available to over 1.8 million UniProtKB accessions.

## INTRODUCTION

The rapid evolution in genetic sequencing over the past decades is leading to an unprecedented growth in the number of protein sequences available in the UniProt Knowledgebase (UniProtKB, http://uniprot.org)—a universal resource for sequence and functional information pertaining to proteins (1). It currently contains over 500 000 manually annotated sequences (UniProtKB/Swiss-Prot) and over 120 million computationally annotated ones (UniProtKB/TrEMBL) despite a near 50% reduction of the size of the holdings in 2015 to remove high sequence redundancy. This increase is set to continue and likely to accelerate even further with the growing appreciation of the role microbiome plays in health and disease. Most of these protein sequences are unlikely to be experimentally characterised and, therefore, they will not be targeted for manual curation. In order to annotate this large protein space, the UniProt team has developed a rule-based prediction system (UniRule) to automatically enrich UniProtKB/TrEMBL proteins with functional annotations. The rules in the UniRule system are manually annotated based on InterPro family classification and experimental annotation in UniProtKB/Swiss-Prot, and then computationally applied to annotate millions of protein sequences in the database (1). Knowledge of protein structure can help elucidate function, and thus enhance computational (and manual) annotations available in UniProtKB.

In parallel to the growth in sequencing data, structural biology has undergone revolutionary changes over the past decade, ranging from dramatic improvements in electron microscopy to wider accessibility and near complete automation of crystallographic techniques. The Protein Data Bank (PDB) is the single global archive of experimentally determined three-dimensional (3D) biomacromolecular structures and associated experimental data (2). It is managed by the Worldwide PDB (wwPDB; http://wwpdb.org) (3), an international consortium, of which the Protein Data Bank in Europe (PDBe; http://pdbe.org) (4) is one of the founding members. PDB receives an increasing number of depositions (over 13 000 in 2017) of ever increasing complexity, yet the pace of growth of the PDB is by necessity slower than that of sequence resources, with increases in coverage of the sequence space proportionate to the increase in the number of PDB entries: from 28 000 unique UniProtKB accessions referenced by 84 000 PDB entries in early 2013 (5) to over 45 000 UniProtKB accessions referenced by over 145 000 PDB entries at present. Robust mechanisms of data discovery and of linking biological contexts pertaining to proteins are essential. A number of resources utilise the structure data from the PDB to annotate protein sequences within related families and superfamilies of sequences (6).

Both the PDBe and UniProtKB are core resources at the European Bioinformatics Institute (EMBL-EBI; http://www.ebi.ac.uk) (7) and within the context of the ELIXIR infrastructure (http://elixir-europe.org) (8). Facilitated by their co-location at EMBL-EBI, the PDBe and UniProt teams developed the Structure Integration with Function, Taxonomy and Sequences (SIFTS) resource (9), which allows for transfer of value-added annotations between the protein sequences and the protein structures, helping to understand mechanisms of protein interactions and function. SIFTS provides residue-level cross-references between protein sequences in UniProtKB and 3D atomic models of those proteins within PDB entries. The resource also collates and distributes residue-level annotations from Pfam (10), InterPro (11), SCOP (12) and CATH (13), and whole sequence level cross-references from IntEnz (14), GOA (15), PubMed (16), and NCBI taxonomy (17), all of which have been part of the SIFTS process as described previously (9). The most recent update added cross-references from Homologene (https://www.ncbi.nlm.nih.gov/homologene) (18) and Ensembl (19), and automatic Pfam assignments based on HMM profiles (20,21). In order to enhance the possibility of transfer of annotations between protein sequences and structures, the underlying SIFTS pipeline was also re-engineered to support multiple cross-references between UniProtKB and PDB, as described below.

The pipeline underlies many features of the PDBe website and REST API (4). Many other bioinformatics resources such as UniProt (1), RCSB PDB (22), PDBj (23), PDBsum (24), Reactome (25), Pfam (10), SCOP2 (26), MobiDB (27) and InterPro (11) rely on the SIFTS resource to establish cross-references between the PDB structures and other biological data in order to serve up-to-date information to their users. From 2018, SIFTS is incorporated into the PDBe Knowledge Base resource (PDBe-KB; http://pdbe-kb.org).

## METHODOLOGY

The basic SIFTS procedure has been described previously (9). Its two main components remain the same: a semi-automated process to identify sequence cross-references from UniProtKB to the protein sequences in the PDB, and a fully automated process to generate residue-level mappings between the two sequences and to add further cross-reference information from other bioinformatics resources. The original procedure was limited to cross-referencing the polypeptide sequence in a given PDB entry to a single UniProtKB accession. This limitation was overcome in the most recent SIFTS infrastructure update by organising the PDB-UniProtKB cross-references into three categories: (i) mapping to a UniProt canonical protein sequence, unchanged compared to the previous implementation, (ii) mapping to all alternative isoforms of the canonical sequence and (iii) mapping to sequences in UniRef90 clusters. The latter two categories will be discussed below.

### Mappings to isoforms

**Figure 1.**
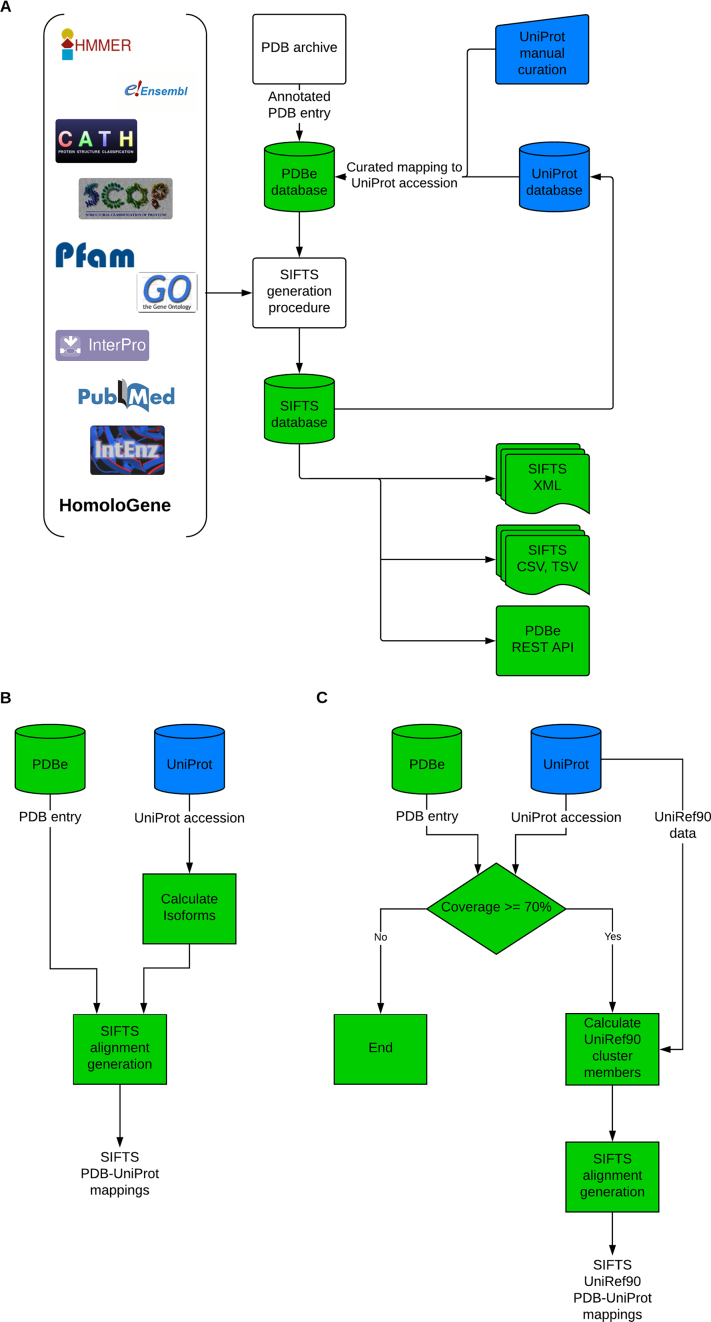
Schematic diagrams of the SIFTS process. (**A**) Overall view of the data flow from PDB, UniProtKB and other resources to data distribution. (**B**) Calculation of direct mappings between protein structures in PDB and UniProtKB sequences, including isoforms. The process in panel B is invoked weekly and the data are released concurrently with the release of new PDB structures (see text). (**C**) Calculation of mappings for UniRef90 dataset. The process in panel C is invoked after the weekly release of new PDB structures.

It is thought that alternative splicing is implicated in a number of diseases, and that nearly all multi-exon protein-coding genes in humans may undergo alternative splicing, giving rise to different isoform protein products (28). One of these products - usually the most prevalent - is termed a ‘canonical’ entry in UniProtKB, and was previously the only option for SIFTS cross-references to protein sequences in the PDB. In order to overcome this limitation, the SIFTS process was updated as follows (Figure 1A and B):

- For each polypeptide sequence in the PDB—the query sequence—retrieve the existing manually annotated cross-reference provided by either the UniProtKB or by the PDB, as described previously (9).
- Expand the set of UniProtKB sequences to be analysed with all the isoforms of the accession from (a), unless the query sequence is identified as a chimeric construct. In the latter case, the set of accessions is not expanded beyond the manually annotated ones.
- Calculate sequence alignments and sequence identity between the query sequence and each UniProtKB accession from the set defined in (b). For canonical UniProtKB sequences, coverage by the PDB sequence is also calculated.
- Annotate the best sequence alignment from (c). Currently, the best alignment is defined as the one with the highest sequence identity with a preference for the canonical accession in the case of a tie.
- Cross-references from Pfam, IntEnz and Homologene are added on the basis of the mappings to the canonical UniProtKB accessions, as these resources do not consider isoform data, while those from Ensembl are added based on the isoform information. Cross-references from GOA, InterPro and preliminary Pfam assignments based on HMM profiles are calculated for the actual query sequence from the PDB.

At the time of writing, 727 unique human proteins (in 2412 PDB entries) have a non-canonical isoform as their best mapping. In total, the PDB archive contains 7202 unique human proteins (in 40 325 PDB entries). Four proteins in seven PDB entries only have valid mappings to non-canonical isoforms (Supplementary Table S1).

The above procedure is integrated into the weekly PDBe release process, and the resulting core SIFTS data are made available publicly along with the weekly PDB release (00:00 UTC each Wednesday). Data are available as a combination of the PDBe REST API (http://www.ebi.ac.uk/pdbe/api/doc/sifts.html), per-entry XML files with residue-level information, and summary flat files in CSV and TSV formats.

### Mappings to UniRef90 clusters

UniProt Reference Clusters (UniRef) are sets of sequences from the UniProtKB, >10 residues in length, that share a level of sequence identity (29) using the CD-HIT algorithm (30). In particular, UniRef90 is built by clustering UniProtKB sequences such that each cluster is composed of sequences that have at least 90% sequence identity to and 80% overlap with the longest sequence (called *the seed sequence*) of the cluster. It is generally expected that proteins belonging to a given UniRef90 cluster are structurally very similar. It is therefore a useful extension to be able to cross-reference UniProtKB accessions to 3D structures in the PDB *via* the UniRef90 clusters. The SIFTS procedure for isoforms described above is applicable for generating mappings to members of UniRef90 clusters with a few configurable modifications (Figure 1C):

- For each polypeptide sequence in the PDB - the query sequence—retrieve the canonical UniProtKB cross-reference (primary accession) from the core SIFTS data, and calculate the coverage of the UniProtKB accession by the query sequence.
- If the coverage from (a) is greater than 70%, retrieve all UniProtKB accessions belonging to the same UniRef90 cluster(s) as the primary accession. For UniRef90 clusters with more than 5000 members, restrict the expanded set to one randomly chosen UniProtKB accession per taxonomy identifier.
- Perform pairwise sequence alignments between the query sequence and the set of UniProtKB accessions from (b), and calculate sequence identity for each alignment.

Currently, additional cross-references from external resources are not included for mappings to UniRef90 clusters. The PDB to UniRef90 mapping procedure currently takes approximately one day to calculate and is thus performed after the weekly release. UniRef90 mapping data become publicly available via the PDBe REST API one week after the PDB data are released.

### Other improvements

Ultimately, the purpose of SIFTS is to provide an infrastructure for transfer of annotations and cross-references between the structure and the sequence domains, represented by the PDB and the UniProtKB data, respectively. Thus, apart from the above improvements, the SIFTS pipeline expanded the coverage of cross-references from other resources through the addition of provisional domain assignments based on Pfam HMM profiles (20), cross-references to Ensembl identifiers and genomic positions (19), Homologene identifiers (18), and additional PubMed cross-references retrieved from UniProtKB. SIFTS continues to include cross-references from GOA (15), InterPro (11), IntEnz (14), CATH (13), SCOP (12) and Pfam (10). For each identified Pfam domain and provisional domain assignment, the coverage by the PDB structure is calculated.

## DATA DISTRIBUTION

Core SIFTS data continues to be distributed as per-entry XML files available from the EMBL-EBI FTP area (ftp://ftp.ebi.ac.uk/pub/databases/msd/sifts/). Their structure remains the same as described previously (9) with the addition of Ensembl genomic position information. Summary information is also distributed as comma- or tab-delimited flat files, also available at the EMBL-EBI FTP tree. Compared to the previous description, three new files were added describing additional mappings:

- Mappings involving only observed PDB residues, i.e., excluding those residues which were present in the experimental sample, but whose atomic coordinates were not modelled (e.g., because of poor electron density) (ftp://ftp.ebi.ac.uk/pub/databases/msd/sifts/csv/uniprot_segments_observed.csv);
- for preliminary Pfam assignments based on HMM profiles (ftp://ftp.ebi.ac.uk/pub/databases/msd/sifts/csv/pdb_chain_hmmer.csv);
- and for Ensembl genomic positions (ftp://ftp.ebi.ac.uk/pub/databases/msd/sifts/csv/pdb_chain_ensembl.csv).

Nearly all of the SIFTS data is also accessible via the PDBe REST API (http://www.ebi.ac.uk/pdbe/api/doc/sifts.html), and some information (e.g. mappings to members of UniRef90 clusters) is only available through this channel. SIFTS data underlie a major part of the PDBe search functionality and the PDB entry pages (4,31).

## APPLICATIONS

The major improvement in the updated SIFTS pipeline is the ability to include multiple mappings between protein sequences found in PDB and UniProtKB entries. The two main applications of this development are the provision of mappings to isoforms and to UniProtKB sequences from UniRef90 clusters.

Including the mappings to members of UniRef90 clusters expands the structural coverage of UniProtKB 40-fold from ∼45 000 UniProt accessions mapped directly to proteins within PDB entries to over 1.8 million UniProtKB accessions with at least 90% sequence identity to structures in the PDB which cover 70% or more of the UniProtKB sequence. Narrowing down to structural coverage of a particular species (Table 1), our analysis shows that while the PDB contains structures of 3010 unique human proteins with at least 70% coverage of the corresponding UniProtKB accession, this expands by 26 673 unique UniProtKB accessions that map to a structure in the PDB via the UniRef90 route. There is considerable redundancy in this set due to a large number (24 056) of unreviewed (TrEMBL) protein isoforms that are included in the UniRef90 clusters, but not in the UniProt human reference proteome (Table 2). The overwhelming majority of these UniProtKB accessions can map to the set of human proteins already present in the PDB, but there are 1318 UniProtKB accessions (970 protein names) for human proteins, which currently only map to a non-human protein structure in the PDB, thus expanding the structural coverage of the human proteome by more than 30%. In the case of the mouse proteome, this expansion more than doubles (from 764 unique protein names to 1954).

**Table 1.** Structure coverage of proteomes of selected model organisms via the UniRef90 clusters

Number of UniProtKB accessions (unique protein names) from an organism → Organism	(1) Direct mappings to PDB entries with at least 70% sequence coverage	(2) In SIFTS UniRef90 datasets, excluding accessions in (1)	(3) In SIFTS UniRef90 datasets, and mapping to a PDB sequence from another organism	(4) In SIFTS UniRef90 datasets, and mapping to a PDB sequence from the same organism	(5) In SIFTS UniRef90 datasets, and mapping to both PDB sequence from the same and from different organism	(6) In SIFTS UniRef90 datasets, and mapping to a PDB sequence from another organism only, i.e., inaccessible from the same species
*Homo Sapiens*	3010 (2959)	26 673 (4918)	1799 (1377)	26 907 (5287)	689 (531)	1318 (970)
*Drosophila melanogaster*	203 (202)	262 (205)	22 (22)	263 (206)	-	21 (21)
*Mus musculus*	764 (752)	4289 (2621)	3264 (2144)	1614 (911)	270 (159)	3045 (1954)
*Escherichia coli* (all subspecies)	2042 (1658)	272 533 (14 080)	27 801 (2307)	258 324 (12 836)	12 925 (1013)	27 663 (2288)
*Saccharomyces cerevisiae* (all subspecies)	1187 (1168)	12 070 (3841)	789 (258)	12 121 (3894)	700 (214)	725 (207)
*Schizosaccharomyces pombe* (all subspecies)	156 (156)	5 (5)	6 (5)	1 (1)	-	4 (4)
*Caenorhabditis elegans*	106 (97)	30 (27)	10 (9)	35 (32)	2 (2)	8 (8)
*Danio rerio*	71 (68)	493 (341)	408 (283)	105 (72)	7 (6)	406 (282)
*Arabidopsis thaliana*	344 (342)	674 (472)	73 (51)	652 (465)	1 (1)	63 (47)
*Triticum aestivum*	48 (48)	396 (118)	279 (81)	134 (49)	12 (8)	276 (79)

**Table 2.** Structure coverage of the UniProt human proteome

		Manually curated human proteins (Swiss-Prot)	Automatically curated human proteins (TrEMBL) and part of the UniProt Reference Proteome	Manually or automatically curated human proteins which are not included in the UniProt Reference Proteome
Number of UniProtKB accessions with a direct SIFTS mapping to proteins in the PDB and with 70% or more sequence coverage	Canonical	2920	1	107
	Other isoforms	2618^a^	8^b^	
Number of UniProtKB accessions in UniRef90 clusters with at least one SIFTS mapping to a PDB structure (excluding direct mappings)	Canonical	240	21	24056
	Other isoforms	169^a^	2279^b^	

^a^The number of isoforms of manually curated proteins (Swiss-Prot) includes an expansion into all isoforms of the canonical sequences from the corresponding row above.

^b^The number of isoforms for mappings (direct or via UniRef90 clusters) to automatically curated proteins (TrEMBL) does not include the expansion of the canonical sequences.

At the time of writing, 27 Enzyme Commission (EC) numbers in the IntEnz database (14), for which no PDB structure is available, map to UniRef90 clusters with at least one PDB entry (Table 3), and thus their structures could potentially be modelled by homology with a degree of confidence. The number of species for which there is at least one protein structure in the PDB is ∼4000, while taking the UniRef90 clusters into account, studies of over 86,000 species (distinct taxonomy identifiers) could benefit from available structure data.

**Table 3.** Enzymes (Enzyme Commission numbers) in the IntEnz resource that are not annotated in the PDB but that belong to UniRef90 clusters with a mapping to PDB structure

Mappings to PDB structures annotated with a different EC number from IntEnz							
EC number in UniRef90	Enzyme name in UniRef90	UniProtKB accession in UniRef90	Sequence identity to PDB entries	PDB entries (possible templates)	EC number associated with PDB entry	Enzyme name in PDB entry	UniProtKB accession mapped to PDB structure
1.1.1.96	Diiodophenylpyruvate reductase	P40925	95%	4mdh 5mdh	1.1.1.37	Malate dehydrogenase	P11708
1.6.2.6	Leghemoglobin reductase	Q41219	96%	1dxl	1.8.1.4	Dihydrolipoyl dehydrogenase	P31023
3.4.24.73	Jararhagin	P30431	95%	3dsl	3.4.24.49	Bothropasin	O93523
3.5.4.45	Melamine deaminase	Q9EYU0	98%	4v1x 4v1y	3.8.1.8	Atrazine chlorohydrolase	P72156
3.7.1.13	2-hydroxy-6-oxo-6-(2-aminophenyl)hexa-2,4-dienoate hydrolase	Q9AQM4	98%	1j1i	3.7.1.8	2,6-dioxo-6-phenylhexa-3-enoate hydrolase	Q84II3
4.1.2.9	Phosphoketolase	Q9AEM9	95%	3ahc 3ahd 3ahe 3ahf 3ahg 3ahh 3ahi 3ahj	4.1.2.22	Fructose-6-phosphate phosphoketolase	D6PAH1
4.2.3.32	Levopimaradiene synthase	H8ZM70	99%	3s9v	4.2.3.18 4.2.3.132	Abieta-7,13-diene synthase Neoabietadiene synthase	Q38710
4.2.3.44	Isopimara-7,15-diene synthase	H8ZM71	92%		5.5.1.12	Copalyl diphosphate synthase	
4.5.1.5	S-carboxymethylcysteine synthase	P0ABK5	100%	5j43 5j5v	2.5.1.47	Cysteine synthase	P0ABK6
5.3.1.34	D-erythrulose 4-phosphate isomerase	Q9ZB26	99%	5ifz	5.3.1.6	Ribose-5-phosphate isomerase	Q8YCV4
6.5.1.6	DNA ligase (ATP or NAD(+))	Q9HHC4	91%	3rr5	6.5.1.1	DNA ligase (ATP)	C0LJI8

Mappings to PDB structures lacking annotation with an EC number from IntEnz							
EC number in UniRef90	Enzyme name in UniRef90	UniProtKB accession in UniRef90	Sequence identity to PDB entries	PDB entries (possible templates)	UniProtKB accession mapped to PDB structure	Unreviewed protein name from mapped UniProtKB accession	
1.14.14.11	Styrene monooxygenase	O50214	100%	3ihm	O33471	Styrene monooxygenase component A	
1.3.1.29	*cis*-1,2-Dihydro-1,2-dihydroxynaphthalene dehydrogenase	P0A170	98%	5xtf 5xtg	G9G7I7	2,3-dihydroxy-2,3-dihydrophenylpropionate dehydrogenase	
1.3.1.60	Dibenzothiophene dihydrodiol dehydrogenase						
2.3.1.228	Isovaleryl-homoserine lactone synthase	Q89VI2	100%	5w8a 5w8c 5w8d 5w8e 5w8g	A0A0N0C224	Autoinducer synthase	
2.3.1.60	Gentamicin 3-*N*-acetyltransferase	P23181	99%	6bvc	Q53396	Aminoglycoside-(3)-*N*-acetyltransferase	
2.4.1.292	GalNAc-alpha-(1→4)-GalNAc-alpha-(1→3)-diNAcBac-PP-undecaprenol alpha-1,4-*N*-acetyl-D-galactosaminyltransferase	Q0P9C5	97%	6eji 6ejj 6ejk	O86151	WlaC protein	
2.8.2.37	Trehalose 2-sulfotransferase	A0QQ53	100%	1tex	P84151	Putative sulfotransferase	
2.8.3.10	Citrate CoA-transferase	P45413	92%	1xr4	Q8ZRY1	Citrate lyase alpha chain	
3.1.1.59	Juvenile-hormone esterase	P19985	100%	2fj0	Q9GPG0	Carboxylic ester hydrolase	
3.2.1.94	Glucan 1,6-alpha-isomaltosidase	Q44052	97%	5awo 5awp 5awq	Q7WSN5	Isomaltodextranase	
3.5.1.105	Chitin disaccharide deacetylase	Q99PX1	99%	3wx7	A6P4T5	Chitin oligosaccharide deacetylase COD1	
4.2.1.163	2-Oxo-hept-4-ene-1,7-dioate hydratase	P42270	100%	2eb4 2eb5 2eb6	Q46982	2-hydroxyhexa-2,4-dienoate hydratase	
4.2.1.168	GDP-4-dehydro-6-deoxy-alpha-D-mannose 3-dehydratase	D3QY10	100%	2gms 2gmu	Q9F118	Putative pyridoxamine 5-phosphate-dependent dehydrase	
4.2.3.108	1,8-Cineole synthase	O81191	92%	2j5c	A6XH05	Cineole synthase	
6.2.1.13	Acetate–CoA ligase (ADP-forming)	Q8U3D6	92%	2csu	O58493	Uncharacterized protein	
6.3.2.39	Aerobactin synthase	Q47318	92%	6cn7	Q6U605	IucA/IucC family siderophore biosynthesis protein	

## CONCLUSION

In conclusion, the SIFTS pipeline was updated to include multiple mappings between the protein structures in the PDB and their sequences in UniProtKB. This allows a more accurate representation of structures of specific isoforms with ∼10% of human proteins in the PDB having their best sequence alignment to a non-canonical sequence in the UniProtKB. More importantly, the expansion of the cross-references to protein sequences in UniRef90 clusters increases the structure coverage of the protein sequence space 40-fold, expanding the applicability of structure-based annotation to over 1.8 million UniProtKB sequences. Inclusion in the SIFTS data of gene IDs and genomic positions from Ensembl enables a more direct cross-referencing of genomic data from PDB structures. SIFTS data are made available via a combination of the per-entry XML files, summary CSV and TSV files and the PDBe REST API.

## Supplementary Material

Supplementary Data
