# Supplementary material for: SIFTS: updated Structure Integration with Function, Taxonomy and Sequences resource allows 40-fold increase in coverage of structure-based annotations for proteins
Source: Nucleic Acids Res. 2018 Nov 16;47(Database issue):D482–9. doi: 10.1093/nar/gky1114 (PMC6324003; doi:10.1093/nar/gky1114)
Supplement: Supplementary Data [file gky1114_supplemental_files.docx]

Supplementary Table S1

Proteins in PDB entries with a cross-reference to non-canonical UniProtKB accessions only.

| PDB code | PDB entity | UniProtKB accession | Protein name | Sequence identity |
| --- | --- | --- | --- | --- |
| 1loi | 1 | P54748-3 | cAMP-specific 3',5'-cyclic phosphodiesterase 4A | 100% |
| 4cgb | 1 | O95834-2  O95834-3 | Echinoderm microtubule-associated protein-like 2 | 100% |
| 4j2x | 2 | P97447-2 | Four and a half LIM domains protein 1 | 100% |
| 5e8n | 3 | Q9D6K9-2 | Ceramide synthase 5 | 100% |
| 5e8o | 2 | Q9D6K9-2 | Ceramide synthase 5 | 89% |
| 5e8p | 3 | Q9D6K9-2 | Ceramide synthase 5 | 89% |
| 5mzm | 3 | Q9D6K9-2 | Ceramide synthase 5 | 89% |
